# Pathogens and Elicitors Induce Local and Systemic Changes in Triacylglycerol Metabolism in Roots and in Leaves of *Arabidopsis thaliana*

**DOI:** 10.3390/biology10090920

**Published:** 2021-09-16

**Authors:** Sebastian Schieferle, Beeke Tappe, Pamela Korte, Martin J. Mueller, Susanne Berger

**Affiliations:** Julius-von-Sachs-Institute for Biosciences, Pharm, Biology, Biocenter, University of Wuerzburg, Julius-von-Sachs-Platz 2, 97082 Wuerzburg, Germany; SebastianSchieferle@web.de (S.S.); beeke.tappe@web.de (B.T.); pamela.korte@uni-wuerzburg.de (P.K.); martin.mueller@biozentrum.uni-wuerzburg.de (M.J.M.)

**Keywords:** triacylglycerols, membrane remodeling, pathogens, elicitors, effectors

## Abstract

**Simple Summary:**

Abiotic and biotic stress conditions result in profound changes in plant lipid metabolism. Vegetable oil consists of triacylglycerols, which are important energy and carbon storage compounds in seeds of various plant species. These compounds are also present in vegetative tissue, and levels have been reported to increase with different abiotic stresses in leaves. This work shows that triacylglycerols accumulate in roots and in distal, non-treated leaves upon treatment with a fungal pathogen or lipopolysaccharide (a common bacterial-derived elicitor in animals and plants). Treatment of leaves with a bacterial pathogen or a bacterial effector molecule results in triacylglycerol accumulation in leaves, but not systemically in roots. These results suggest that elicitor molecules are sufficient to induce an increase in triacylglycerol levels, and that unidirectional long-distance signaling from roots to leaves is involved in pathogen and elicitor-induced triacylglycerol accumulation.

**Abstract:**

Interaction of plants with the environment affects lipid metabolism. Changes in the pattern of phospholipids have been reported in response to abiotic stress, particularly accumulation of triacylglycerols, but less is known about the alteration of lipid metabolism in response to biotic stress and leaves have been more intensively studied than roots. This work investigates the levels of lipids in roots as well as leaves of *Arabidopsis thaliana* in response to pathogens and elicitor molecules by UPLC-TOF-MS. Triacylglycerol levels increased in roots and systemically in leaves upon treatment of roots with the fungus *Verticillium longisporum*. Upon spray infection of leaves with the bacterial pathogen *Pseudomonas syringae*, triacylglycerols accumulated locally in leaves but not in roots. Treatment of roots with a bacterial lipopolysaccharide elicitor induced a strong triacylglycerol accumulation in roots and leaves. Induction of the expression of the bacterial effector AVRRPM1 resulted in a dramatic increase of triacylglycerol levels in leaves, indicating that elicitor molecules are sufficient to induce accumulation of triacylglycerols. These results give insight into local and systemic changes to lipid metabolism in roots and leaves in response to biotic stresses.

## 1. Introduction

Abiotic and biotic environmental factors have great impact on all aspects of plant life and metabolism. In general, adverse conditions and pathogen infection negatively affect plant growth and reproduction [1]. In addition, several primary and secondary metabolites are synthesized to cope with these stresses and ameliorate their adverse effects [2]. This is reflected by profound changes in plant metabolism. Alterations in lipids comprise degradation and synthesis of membranes. Remodeling of the lipid composition of membranes has been shown to be important for adaptation to the environmental conditions [3,4]. In addition to membrane lipids, the levels and composition of triacylglycerols (TGs) are also changed. TGs are involved in diverse aspects of plant life. As storage compounds, TGs provide energy and carbon skeletons during early seedling growth [5,6]; they also play a role in flower development and are present in vegetative tissue [6]. Occurrence of and changes to TG levels in leaves have especially been reported. In leaves, TGs are mainly found in lipid droplets in the cytosol or in plastoglubules in plastids. An increase in the amount of leaf oil bodies has been reported during senescence [7]. Accumulation of TGs has been described in different plant species in response to several abiotic stresses (see review by [8]) such as nitrogen and phosphate deprivation, heat, and drought stress [9,10,11]. Even though the function of TG accumulation in stress responses is not clear yet, there are indications that the remodeling of membranes associated with an accumulation of TGs positively affect adaptation to or tolerance of these abiotic stresses [12,13]. *Arabidopsis* lines with increased levels of TGs in leaves are more sensitive to elevated temperatures [14] and lines defective in the enzyme phospholipid:diacylglycerol acyltransferase 1 (PDAT1) are also more sensitive to heat [15]. 

Less is known on the connection between TGs and biotic stress. TGs accumulate in *Arabidopsis* plants treated with an avirulent strain of *Pseudomonas syringae* [16]. There are also indications that the TG content of leaves affects interaction with pests. Feeding of *Arabidopsis* lines containing higher basal levels of TGs to *Trichoplusia ni* resulted in more weight gain in the herbivorous insects [14]. Furthermore, a role for TGs in plant defense was proposed. Oil bodies containing a dioxygenase and a peroxidase at their surface accumulate after infection with the fungus *Colletotrichum higginsianum* and in senescing leaves. These enzymes can produce 2-hydroxy-octadecatrienoic acid from α-linolenic acid. 2-hydroxy-octadecatrienoic acid displays antifungal activity, indicating that oil bodies are involved in producing a phytoalexin [7,17]. Since in all these experiments only leaves were tested, accumulation in different organs such as roots remains to be addressed. 

Elicitors are molecules which can elicit defense and general stress responses. These substances comprise proteins, peptides, lipids and oligosaccharides and can originate from the plant, interacting microbes, herbivores or the abiotic environment [18]. Several elicitors from bacteria and fungi are known [19]. These molecules induce pattern-triggered immunity. Lipopolysaccharides (LPS) are microbe-associated molecular patterns (MAMPs) which are present in the outer membrane of gram negative bacteria and can stimulate immunity in a broad range of eukaryotes [20]. Activation of immune responses by LPS in plants has also been reported [21,22].

Another layer of plant immunity is based on the perception of microbe-derived effector molecules, also termed race-specific elicitors, which induce effector-triggered immunity. According to established models, these effectors are virulence factors secreted by the pathogen to overcome plant defense mechanisms. Resistance proteins of the host plant recognize the presence or activity of these effectors, resulting in very fast and strong activation of defense responses [23].

Even though many metabolic responses to fungal and bacterial pathogens as well as elicitors have been described, little is known about their effect on TG metabolism. In addition, TG metabolism in roots in response to biotic stress remains elusive. To better understand the connection between TGs and biotic stress and to expand knowledge on root responses to biotic stress, we used a hydroponic system which enabled us to analyse lipid metabolism in roots as well as in leaves. This work investigated the alteration in lipid metabolism in roots in comparison to leaves in response to the fungal pathogen *Verticillium longisporum* (*V.l.*) and the bacterial pathogen *P. syringae*, as well as to LPS as a bacteria-derived general elicitor and to the AVRRPM1 protein as a bacteria-derived effector.

## 2. Materials and Methods

### 2.1. Plant Cultivation

*Arabidopsis thaliana* Col-0 was used as a wild type. The transgenic line Dex::avrRPM1 expressing the AVRRPM1 effector protein with HA-tag under the control of dexamethasone-inducible system was kindly provided by Geng and Mackey [24]. The mutants *pdat1-2* (SALK_065334c) and *dgat1-1* (CS3861) were kindly provided by Changcheng Xu [25].

Cultivation was performed according to [26]. Surface sterilized seeds were placed on 0.5 mL micro tubes filled with the standard nutrient solution described in [27] with 1% Phyto Agar (Duchefa) and left for 2 days (d) at 4 °C in darkness. Subsequently, plants were grown in a plant cabinet at 20 °C under a 9 h photoperiod (80 µmol photons m^−2^·s^−1^). After 14 d, 2 mm of the bottom of the micro tube was removed and the tube placed in a hole in the lid of sterile 50 mL tubes containing sterile liquid standard nutrient solution. The 50 mL tubes were covered with aluminium foil to protect the roots from light. Plants were cultivated for an additional 4 weeks in the same conditions. One week before harvest, the nutrient solution was refilled.

### 2.2. Pathogen Treatment

*Verticillium longisporum* isolate *Vl43* (*V.l.*) was essentially cultivated and harvested as described in [28] for 10 d in potato dextrose broth containing 50 µg/mL cefotaxime on a shaker in darkness. Before infection, the medium was exchanged for sporulation medium (Czapek–Dox-medium containing 50 µg/mL cefotaxime) and cultivated for another 4 d. Conidiospores were harvested using a sterile whatman filter and diluted with a hydroponic medium to 5 × 10^6^ spores/L. For treatment, roots were placed in new 50 mL tubes containing the hydroponic medium with spores or, for the control plants, without spores.

The avirulent strain *Pseudomonas syringae* pv. *tomato* DC3000 avrRPM1 was grown overnight at 28 °C on a shaker in KingsB medium containing 50 mg/L rifampicine and 5 mg/L tetracycline. Bacteria were pelleted by centrifugation for 10 min. The pellet was washed two times with 10 mM MgCl_2_ and adjusted to OD_600_ of 0.5. Silwett L77 was added to a concentration of 0.04%. For the treatment, rosettes were densely sprayed on the adaxial and abaxial sides until the whole surface was covered with the liquid.

### 2.3. Elicitor and Effector Treatment

A solution of 10 mg/mL lipopolysaccharide from *Pseudomonas aeruginosa* (Sigma, Steinheim, Germany, L9143) in water was prepared. Root treatment was performed by adding the stock solution to the hydroponic medium to a final concentration of 50 µg/mL. 

Dex::avrRPM1 plants were treated by spraying the leaves with a final concentration of 2 µM dexamethasone containing 0.005% Silwett L77. 

### 2.4. Quantification of V.l.

For the isolation of DNA, 400 µL of extraction buffer (2% cetyltrimethylammonium bromide, 1.4 M NaCl, 20 mM EDTA, 0.1 M Tris/Cl pH 8) was added to 100 mg homogenized leaf material. After incubation for 15 min at 65 °C 400 µL chloroform/isoamylalcohol 24:1 (*v/v*) were added. The mixture was shortly centrifuged and the upper phase was collected. DNA was precipitated by adding an equal volume of isopropanol. The DNA pellet was washed twice with 70% ethanol, dried, and dissolved in water. The concentration was determined spectrophotometrically and adjusted to 50 µg/mL. The amount of fungal DNA was determined by qPCR using 2 µL of the DNA template with *V.l.* specific primers (Fwd: 5′ CAgCgAAACgCgATATgTAg 3′; Rev: 5′ggCTTgTAgggggTTTAgA 3′) for amplifying a specific *V.l.* fragment encoding parts of the 5S rRNA, and calculated relative to plant actin 2/8 (Fwd: 5′ ggTTTTCCCCAgTgTTgTTg 3′; Rev: 5′ CTCCATgTCATCCCAgTTgC 3′). 

### 2.5. Lipid Analysis

Roots and shoots were separately harvested after the time points indicated, flash frozen in liquid nitrogen, homogenized with pestle and mortar and stored at −80 °C. Roots or the entire rosette of two plants were combined for one sample (except for the data for Supplemental Table S1, where individual plants were used). 50–55 mg of fresh plant material (corresponding to approximately 70% of the harvested root material or 40% of the leaf material, respectively) was lyophilized, with the dry weight determined and used for calculation since the water content of leaves of pathogen treated plants can be lower than of control plants. The dried plant material was extracted with 1 mL chloroform/methanol (3:2, *v:v*) using a mixer mill at 21 Hz for 10 min. The extraction solvent contained 1 µg/sample of MGDG 36:0, DGDG 36:0 and PC 34:0 and 0.1 µg/sample of TG 30:0 as internal standards. After addition of 200 µL water a second homogenization step was performed, again using a mixer mill at 21 Hz for 10 min. The samples were centrifuged for phase separation and the organic phase containing the lipids was evaporated in a vacuum concentrator at 45 °C. The residue was dissolved in 50 µL isopropanol and mixed thoroughly. Insoluble compounds were removed by centrifugation and the supernatant was used for subsequent Ultra Performance Liquid Chromatography coupled with mass spectrometry (UPLC-MS) analysis. Measurement and quantification of lipids was performed as described in [9]. For chromatographic separation and detection an Acquity Ultra Performance Chromatography system coupled to a Synapt G2 HDMS quadrupole time-of-flight hybrid mass spectrometer was used (all Waters). Compounds were separated using a BEH C18 column (2.1 × 100 mm × 1.7 µm) equipped with a VanGuard pre-column (2.1 × 5 mm × 1.7 µm) and an Acquity column in-line filter. A linear binary solvent gradient of 30–100% of eluent B over 15 min at a flow rate of 0.3 mL/min was used, in which eluent A consisted of 10 mM ammonium acetate in water:acetonitrile (60:40, *v/v*) and eluent B of 10 mM ammonium acetate in isopropanol:acetonitrile (90:10, *v/v*). Analytes were detected as ammonia adducts for TGs [M + NH4]^+^, MGDGs [M + NH4]^+^ and DGDGs [M + NH4]^+^. PC were detected as protonated PCs [M + H]^+^. The electrospray ionisation source (ESI) was operated in positive mode at 120 °C with a capillary voltage set to 1.8 kV, a cone voltage of 25 V and nitrogen as desolvation gas (350 °C, flow rate: 800 L/h^−1^). The HR (high resolution) data were acquired over the mass range of 50–1200 D. The acquisition and processing of the chromatograms was performed using MassLynx and QuanLynx (version 4.1; all Waters).

### 2.6. Statistical Analysis

For statistical analysis of the differences between treatment and control or between the mutant and control lines, Students *t*-test was used.

## 3. Results

### 3.1. TGs Accumulate upon Root Infection with Verticillium in Roots and Systemically in Leaves

**Figure 1:**
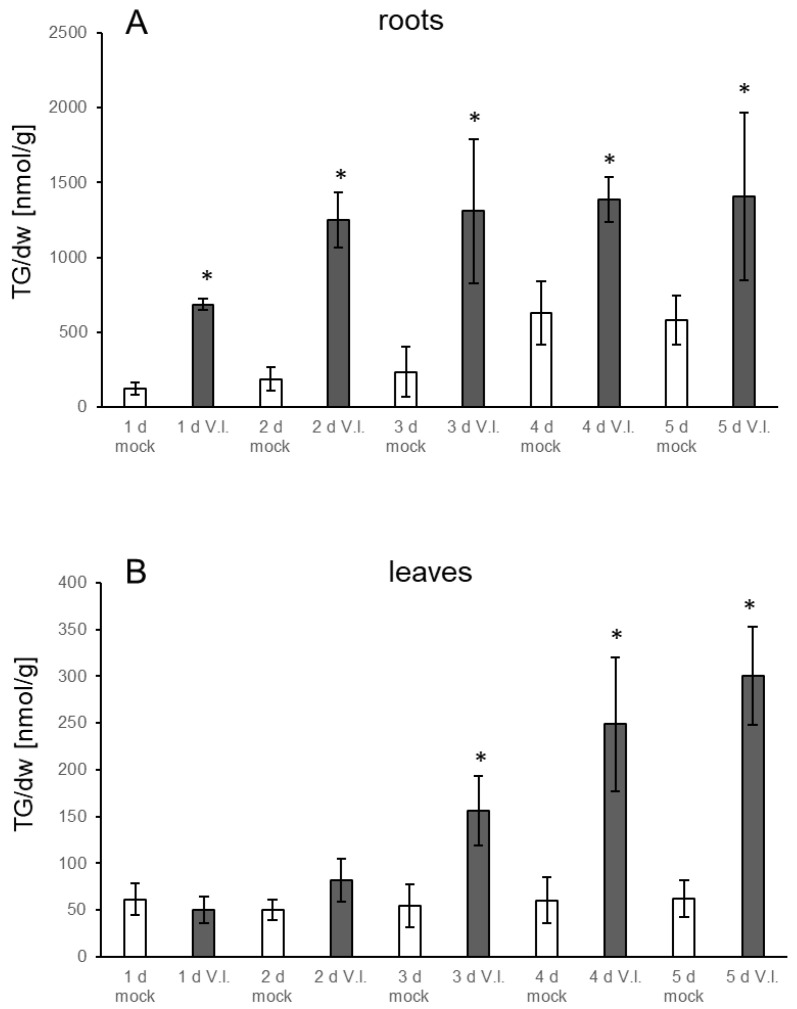
Accumulation of TGs in (**A**) roots and (**B**) leaves of Col-0 in response to treatment of roots with *V.l.* (dark grey bars) or mock (white bars) at different time points. Shown is the mean of five biological replicates ± sd of the sum of TGs [nmol/g dry weight] with 52 and 54 acyl carbon atoms and one to nine double bonds. Asterisks indicate significant differences between the *V.l.* treated samples and the corresponding mock treated sample (* *p* < 0.05).

TG levels increase in response to several stresses in leaves. Very prominent accumulation was, e.g., found upon heat treatment of seedlings as well as upon infection of leaves with *P. syringae* [9,16]. However, less is known about levels in roots. Therefore, we established a system for biotic stress which enables analysis of lipid metabolism in roots. *Verticillium* is a fungal pathogen that infects roots and migrates through the vascular system to the leaves. Plants were grown in a hydroponic medium and treated by adding a spore suspension of *V.l.* to the medium surrounding the roots. Leaves and roots were harvested at different time points. The first symptoms were visible on leaves on day three post inoculation: leaves started to turn red and over the following days wilting was observed. Along the roots, there was obvious proliferation of *V.l.* mycelium. TG levels in the leaves of mock treated control plants did not change during the different time points, while a slight increase in the roots could be observed which was significant at day four (Figure 1). Notably, TG levels were considerably higher in roots of mock treated plants than in leaves, e.g., on days two and three the TG content in roots was around 200 nmol/g dry weight (dw) while the level in leaves was around 50 nmol/g dw. In response to the *V.l.* inoculation, TG levels in infected roots were already higher one day after infection compared to the mock treated plants, showing a clear further strong increase at day two. There was a further minor increase on the following days, reaching the maximum at day four with 1387 nmol/g dw. TG levels were 6.7-fold and 5.6-fold higher compared to the control plants on day two and day three, respectively. In leaves of pathogen treated plants, TGs started to accumulate on day two and a significant increase was detectable at day three. Maximum levels were observed on day five with 301 nmol/g dw, corresponding to a five-fold higher level compared to the control plants. This shows that *V.l.* causes an accumulation of TGs in treated roots and, in addition, in distal non-treated leaves of inoculated plants. Since Verticillium has been shown to be able to migrate via the vasculature system, the TG accumulation in the leaves might have been induced after the fungus reached the leaves. However, qPCR analysis of Verticillium DNA in leaves showed only low levels of fungal DNA at all time points. No significant increase of fungal DNA even after four and five days in comparison to day one was detectable (Supplemental Table S1). In some leaves the fungus was below the detection level, but these leaves still accumulated TGs (Supplemental Table S1). A correlation analysis showed that TG levels do not correlate with the amount of Verticillium in leaves and are independent of the presence of this fungus (Supplemental Figure S7). This indicates a systemic TG accumulation in the leaves after treatment of the roots.

For detailed analyses, the 3 d time point was chosen because at this time point TG levels had strongly increased in leaves and roots and visible symptoms were only minor.

**Figure 2:**
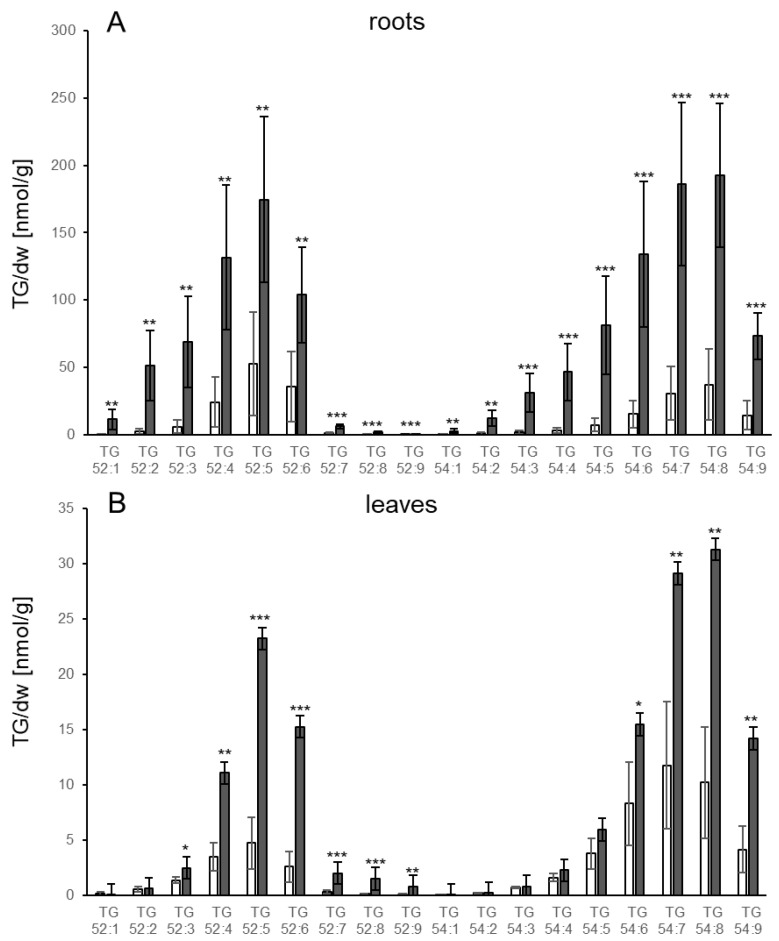
Accumulation of different TG species in (**A**) roots and (**B**) leaves of Col-0 3 d after treatment of roots with *V.l.* (dark grey bars) or mock (white bars). TG species are characterized by their total number of acyl carbons and the number of double bonds. TG levels [nmol/g dry weight] shown represent the mean of five biological replicates ± sd. Asterisks indicate significant differences between the *V.l.* treated samples and the corresponding mock treated sample (* *p* < 0.05; ** *p* < 0.01; *** *p* < 0.001).

The changes in lipid levels were analysed comprehensively. The TGs were examined with regard to their FA composition. In the roots and leaves of control plants the pattern of TG species was similar overall, with small differences. The TG species with the highest basal levels were 52:5 in roots and 54:7 in leaves, respectively (Figure 2). More pronounced differences between leaves and roots were detected after pathogen treatment. Certain TG species containing fatty acids (FA) with one or two double bonds (52:2, 52:3, 54:2, 54:3, 54:4, 54:5) increased significantly in roots while in leaves these TG species showed no or only a less than twofold increase (52:3). In leaves, the most abundant TG species after *V.l.* treatment were the TG species 54:8, 54:7 and 52:5. In neither roots nor leaves was there very much accumulation of 52:9.

**Figure 3:**
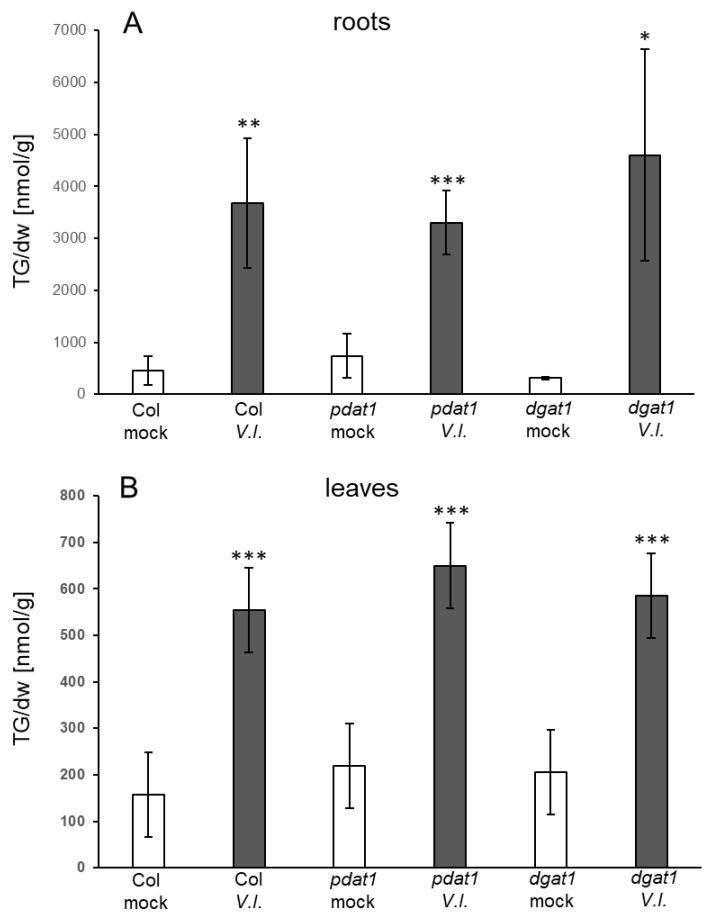
Accumulation of TGs in (**A**) roots and (**B**) leaves of Col-0, *pdat1-2* and *dgat1-1* plants in response to treatment of roots with *V.l.* (dark grey bars) or mock (white bars) at day 3. Shown is the mean of five biological replicates ± sd of the sum of TGs [nmol/g dry weight] with 52 and 54 acyl carbon atoms and one to nine double bonds. Asterisks indicate significant differences between the *V.l.* treated samples and the corresponding mock treated sample (* *p* < 0.05; ** *p* < 0.01; *** *p* < 0.001). There was no significant difference between the mutant lines and the wild type in the corresponding samples.

In a different approach, mutants in TG biosynthesis were analysed to address the question which biosynthetic pathways were used. The enzyme PDAT catalyses the transfer of FA from phosphatidylcholines (PCs) on diacylglycerols (DGs) and prefers polyunsaturated FAs as substrates, while the enzyme diacylglycerol acyltransferase (DGAT) uses the acyl-CoA-pool and preferentially transfers FAs with few or no double bonds to DGs [29]. The accumulation of TGs in response to *V.l.* treatment was analysed in the mutants *pdat1-2* and *dgat1-1* in comparison to the wild type. TG levels in the control samples of all three genotypes were similar and did not show significant differences. Upon *V.l.* treatment, TG levels in the roots and leaves of the mutants *pdat1-2* and *dgat 1-1* increased significantly. There was no significant difference in the TG accumulation in leaves and roots of the mutants compared to the wild type (Figure 3). This indicates that neither PDAT1 nor DGAT1 is essential for TG accumulation in response to *V.l.*

In order to analyse whether levels of additional lipids are altered, the levels of DGs, PCs, diacylgalactosyldiacylglycerols (DGDGs) and monogalactosyldiacylglycerols (MGDGs) were also determined. Only small alterations in the levels of DGs were observed. Overall, DGs did not decrease; rather, in roots there was a small increase which was not significant with respect to total DGs (Supplemental Figure S1). Similarly, no decrease of PC levels in roots or leaves was detected (Supplemental Figure S2). MGDGs and DGDGs are the predominant lipids in plastid membranes. This is reflected by much higher levels of both lipid classes in leaves compared to roots (Supplemental Figures S3 and S4). There was no strong change in MGDG levels in roots or leaves (Supplemental Figure S3). DGDG levels in leaves did not change. In roots there was a decrease in 36:6, and non-significant increases of some DGDGs (Supplemental Figure S4). In the case of massive degradation of membranes, levels of MGDG, DGDG and PCs would be expected to decrease. The results indicate that there is no major degradation of membranes 3 d after *V.l.* treatment.

### 3.2. TG Accumulation in Response to P. syringae 

**Figure 4:**
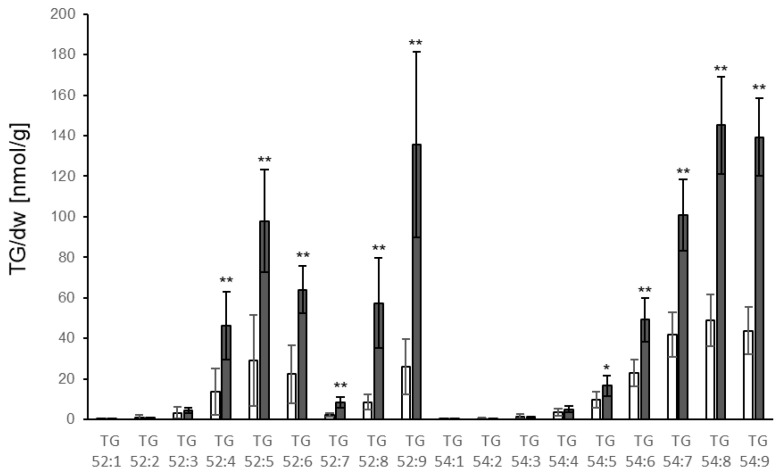
Accumulation of different TG species in Col-0 leaves 1 d after spraying of leaves with *P. syringae* (dark grey bars) or mock (white bars). TG species are characterized by their total number of acyl carbons and the number of double bonds. TG levels [nmol/g dry weight] shown represent the mean of five biological replicates ± sd. Asterisks indicate significant differences between the *P. syringae* treatment and the mock treatment (* *p* < 0.05; ** *p* < 0.01).

It has been reported that TGs accumulate in response to treatment with *P. syringae* [16]. In order to compare changes in lipid metabolism between different pathosystems, leaves of hydroponically grown plants were sprayed with a suspension of *P. syringae*. Symptoms were visible 1 d after treatment when leaves and roots were harvested and analysed. As expected, TG levels increased in leaves upon treatment, with a threefold significant increase of total TGs. Levels of the TG species are shown in Figure 4. In contrast to the *V.l.* system, *P. syringae* infection resulted in a very strong accumulation of TG 52:9. Additionally, TG 54:8 and 54:9 showed a strong increase. No increase could be detected of TGs with only one or two double bonds within the FA chain, suggesting that de novo synthesized FAs do not substantially contribute to the FA pool used for TG synthesis upon *P. syringae* treatment. In contrast to the *V.l.* system, no increase in TG levels could be detected in distal tissue (roots, Supplemental Figure S5). This indicates that *P. syringae* infection of leaves induces only local alterations in leaf TG metabolism, and no systemic changes in roots.

### 3.3. TG Accumulation Can Also Be Induced by Elicitors

**Figure 5:**
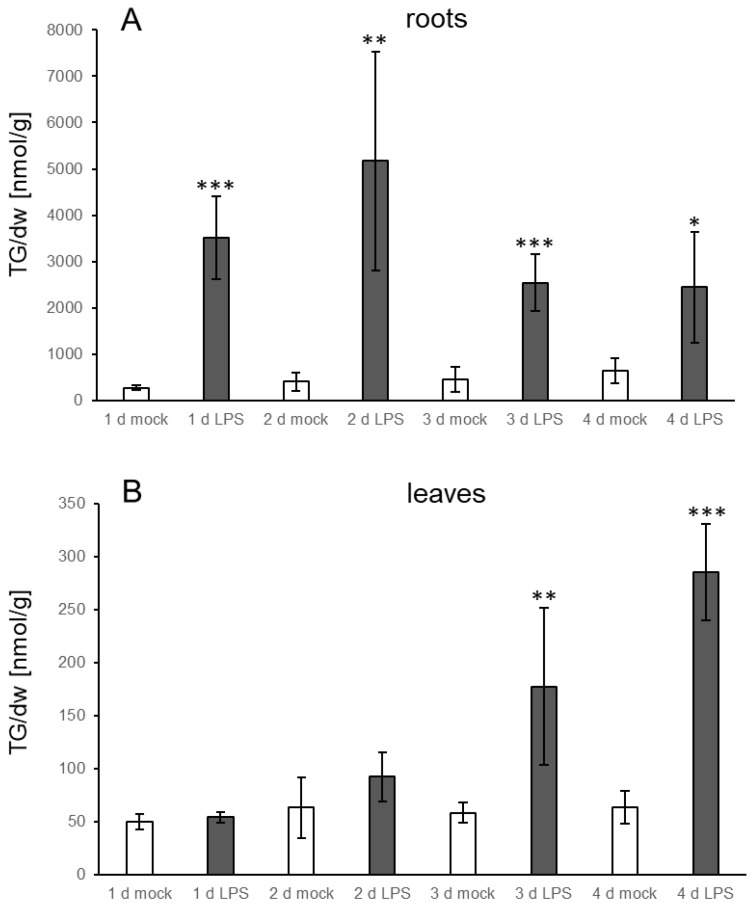
Accumulation of TGs in (**A**) roots and (**B**) leaves of Col-0 in response to treatment of roots with 50 µg/mL LPS (dark grey bars) or mock (white bars) at different time points. Shown is the mean of five biological replicates ± sd of the sum of TGs [nmol/g dry weight] with 52 and 54 acyl carbon atoms and one to nine double bonds. Asterisks indicate significant differences between the LPS treated samples and the corresponding mock treated sample (* *p* < 0.05; ** *p* < 0.01; *** *p* < 0.001).

**Figure 6:**
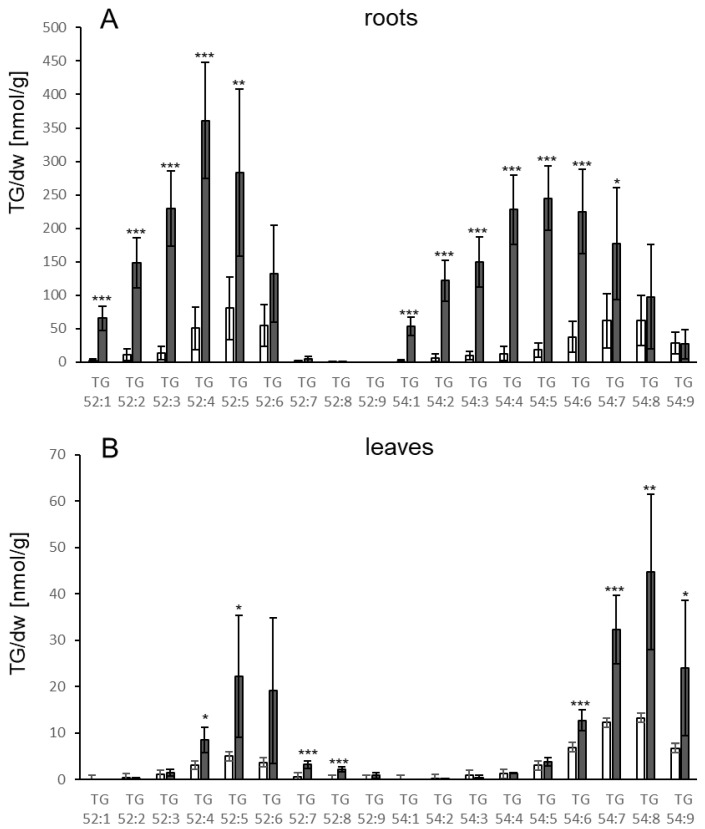
Accumulation of different TG species in (**A**) roots and (**B**) leaves of Col-0 3 d after treatment of roots with 50 µg/mL LPS (dark grey bars) or mock (white bars). TG species are characterized by their total number of acyl carbons and the number of double bonds. TG levels [nmol/g dry weight] shown represent the mean of five biological replicates ± sd. Asterisks indicate significant differences between the LPS treated samples and the corresponding mock treated sample (* *p* < 0.05; ** *p* < 0.01; *** *p* < 0.001).

Since several MAMPs have been reported to elicit similar defense responses as whole living pathogens [19], LPS from *Pseudomonas aeruginosa* was used to test whether addition of the elicitor to roots in the hydroponic system results in TG accumulation. As shown in Figure 5A, TG levels already increased in roots significantly at day one, showing a further increase at day two (12.6-fold) to approximately 5000 nmol/g dw. At the following days, TG levels slightly declined but were still significantly elevated in comparison to the control treatment. Increases were also detectable in leaves, but at later time points compared to the treated roots. At day three, TG levels in LPS treated leaves were significantly higher than the levels in mock treated leaves and reached a maximum at day four (285 nmol/g dw; 4.5-fold increase) (Figure 5B). This indicates that the presence of an elicitor is sufficient to induce TG accumulation locally and systemically. The pattern of TG species in leaves and roots upon LPS treatment (Figure 6) is similar to the pattern observed upon treatment with *V.l.* (Figure 2).

### 3.4. TG Accumulation Can Also Be Induced by Effectors 

**Figure 7:**
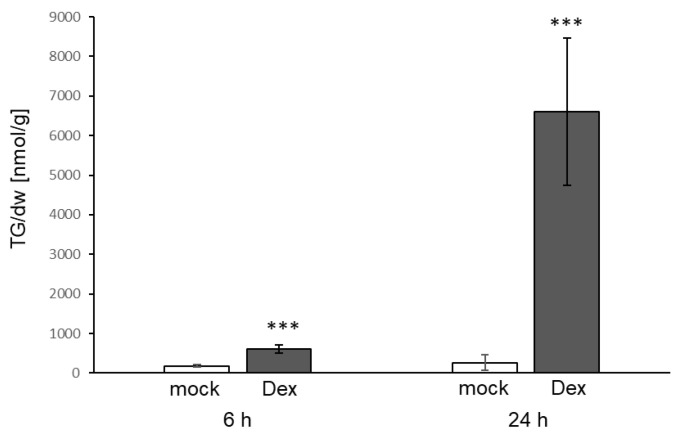
Accumulation of TGs in leaves of Dex::avrRPM1 plants 6 h and 24 h after spraying of leaves with dexamethasone to induce expression of AVRRPMI (Dex, dark grey bars) or mock (white bars). Shown is the mean of at least four biological replicates ± sd of the sum of TGs [nmol/g dry weight] with 52 and 54 acyl carbon atoms and one to nine double bonds. Asterisks indicate significant differences between the dexamethasone treated samples and the corresponding mock treated sample (*** *p* < 0.001).

**Figure 8:**
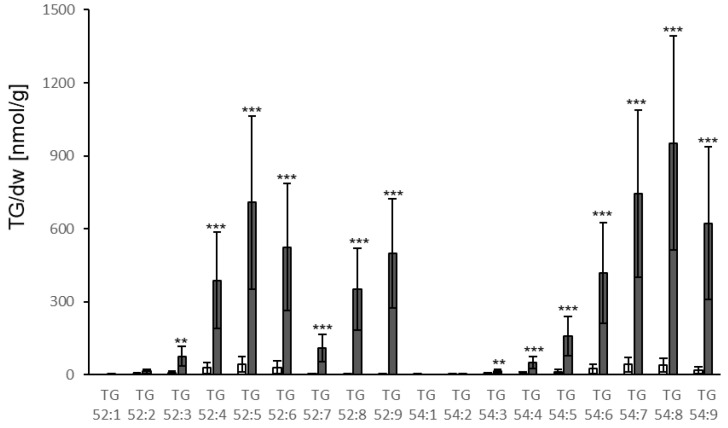
Accumulation of different TG species in leaves of Dex::avrRPM1 plants 24 h after spraying of leaves with dexamethasone to induce expression of AVRRPMI (Dex, dark grey bars) or mock (white bars). TG species are characterized by their total number of acyl carbons and the number of double bonds. TG levels [nmol/g dry weight] shown represent the mean of at least four biological replicates ± sd. Asterisks indicate significant differences between the dexamethasone treated samples and the corresponding mock treated sample (** *p* < 0.01; *** *p* < 0.001).

Effector molecules of *P. syringae* elicit a strong and fast immune response resulting in resistance when the effector or its activity is detected by the corresponding plant resistance protein [30]. In order to investigate whether TG accumulation also occurs upon the recognition of a *P. syringae* effector, a plant line expressing the bacterial AVRRPM1 protein under the control of a dexamethasone-inducible promoter was used. Spraying of leaves with dexamethasone led to very strong TG accumulation in leaves. Levels in the leaves reached 6601 nmol/g dw 24 h after dexamethasone treatment which corresponds to an 11-fold increase compared to the levels at 6 h and a 25-fold increase compared to the control treatment (Figure 7). Similar to the response to *P. syringae* infection, the strongest accumulation was observed for TGs containing polyunsaturated FAs, particularly TG species 54:8, 54:9, 54:7, 52:9 and 52:5 (Figure 8). No increase of TG levels was detected in the roots (Supplemental Figure S6).

## 4. Discussion

### 4.1. TG Levels Change in Response to Biotic Stress in Roots

Changes in the levels of different lipids in response to unfavourable conditions have been reported. Here, we investigated the changes in lipid levels upon treatment with the fungal pathogen *V.l.*, the bacterial pathogen *P. syringae* and two defense-eliciting molecules from Pseudomonas strains. Since profound changes were mainly found in TG levels, we focused on the more detailed study of this lipid class. 

While factors determining the seed oil content and composition have been intensively studied, less is known about TGs in vegetative tissue [6]. In leaves, accumulation of TGs in response to different stresses has been reported. Among abiotic factors, the stress conditions heat, drought and cold treatment resulted in approximately 6-fold, 2.5-fold and 8-fold TG accumulation in leaves, respectively [9,31]. Regarding biotic stress, an increase in the amount of TGs in leaves upon inoculation with the bacterial pathogen *P. syringae* of around 9-fold and of lipid droplets associated with the enzyme DOX1 in the interaction with the fungus *C. higginsianum* of more than 100-fold were reported [16,17]. 

Little is known about TG accumulation in roots. This prompted us to determine TG levels in roots as well as leaves in response to biotic stress factors. Treatment of roots with the fungal pathogen *V.l*. or with the bacterial-derived MAMP LPS increased TG levels 6.7 fold and 12.6 fold in roots, respectively. This shows that root tissue also responds to biotic stress factors with TG accumulation. These increases are considerably higher than the increases due to abiotic stresses reported so far in roots. Phosphate starvation resulted in a 2.5 fold increase of TG levels in tomato roots and salt stress enhanced TG levels in the sweet potato variety Xu 32 by a factor of 1.4 [32,33]. In most cases of TG accumulation in leaves, this was reported to occur mainly in cytosolic lipid droplets rather than plastid localized plastoglobules [6]. Therefore, it is likely that roots also store the accumulating TGs in lipid droplets in the cytosol.

### 4.2. Bacterial Elicitors and Effectors Induce Strong Accumulation of TG

The presence of microbe-derived eliciting molecules is sufficient for the induction of several responses to pathogens. For the bacterial microbe Pseudomonas, MAMPs as well as effector proteins are known. Therefore, we analysed whether the accumulation of TGs can be induced by these molecules, as it was by *P. syringae* infection.

Treatment of leaves with *P. syringae* led to a moderate (around three-fold) increase in TG levels. The levels were much lower than the levels reported in [16] after *P. syringae* treatment. This might be due to the much higher bacterial load and the different treatment method used by [16]. In addition, the work of Zoeller et al. focused on oxidized lipids, which showed a more drastic increase than total TGs did. LPS is a MAMP derived from gram-negative bacteria which activates responses in Arabidopsis such as increase in calcium levels, induction of gene expression, increase in ROS levels and MAPK activation [21]. Analysing the effect of LPS treatment of roots on TG levels revealed a strong increase in roots and leaves. The effect of LPS in leaves was stronger (4.5-fold increase) compared to the effect of treatment with bacteria, even though LPS was applied to roots and bacteria were inoculated on leaves. In addition to MAMPs, the avirulent Pseudomonas strain that was used also contains effector proteins such as AVRRPM1. Expression of the AVRRPM1 protein in plants induced the strongest increase of all treatments tested here, reaching the highest TG levels in leaves (6601 nmol/g dw). This indicates that both bacterial compounds are potent elicitors of TG accumulation in Arabidopsis. 

The receptors for elicitors/effectors used in this study are known. LPS and in particular 3-hydroxy decanoic acid are recognized by Lipopolysaccharide-Specific Reduced Elicitation (LORE) [21], while the AVRRPM1 effector is sensed by the R-protein RPM1 [30]. Furthermore, several downstream components of the signal transduction chains are known. It will be important in further studies to elucidate which of these signaling factors are involved in the accumulation of TGs in response to pathogens and elicitors.

### 4.3. Verticillium and LPS Also Induce Increases in TG Levels in Distal Leaves

The data presented here show that the fungal pathogen *V.l.* and the MAMP LPS induce TG accumulation in treated roots as well as in distal, untreated leaves. Increases in leaves were around five-fold. Since TG accumulation in leaves occurs later than in roots, a spreading of the fungus through the vascular tissue might be the reason for the increase in leaves. However, no correlation between the fungal DNA amount and TG accumulation was found at the time points examined, nor was a significant increase of fungal DNA. This suggests that TG accumulation was induced systemically after root infection. A long-distance signal in the Arabidopsis-Verticillium system has been proposed based on the fact that the receptor for jasmonic acid isoleucine Coronatine Insensitive 1 (COI1) is only required in the root in order for more proliferation of the fungus in leaves to occur [28]. In addition, the fact that the elicitation of TG accumulation in leaves in response to LPS treatment of roots did not depend on the presence of living pathogens indicates the presence of root to shoot signaling. Elicitors have also been identified for fungal pathogens and particularly for *Verticillium daliae* [34,35,36], and related proteins of *V.l.* might contribute to the induction of TG synthesis. Remarkably, all root treatments (either with *V.l.* or with LPS) resulted in TG accumulation in leaves, while none of the leaf treatments (*P. syringae* or AVRRPM1 expression) led to increased TG levels in roots. This could be explained either by only the root being capable of generating the signal(s), or by transport of the signal(s) through the xylem. Which signals and mechanisms are responsible for this root to shoot signaling is an exciting question which needs still to be elucidated. 

### 4.4. Which Pathways Contribute to TG Synthesis in Response to Fungal Infection?

The enzymes and pathways involved in TG biosynthesis have been well studied and comprehensively reviewed recently [29]. FAs for TG synthesis can either originate from remodeling/degradation of membranes or from de novo synthesis [29]. The enzymes and pathways which contribute to TG synthesis determine the FA composition of TGs. The majority of FAs which are derived from membrane lipids are polyunsaturated. The enzyme PDAT1 catalyses the transfer of FA from the *sn-2* position in PCs on DGs and prefers polyunsaturated FAs as substrates [29]. The resulting lyso-PC can be converted to PC by incorporation of an FA from the acyl-CoA-pool. FAs synthesized de novo in plastids are dominated by FAs with no or few double bonds, particularly 18:1, 16:0 and 18:0 [29,37]. The enzyme DGAT1 uses the acyl-CoA-pool and preferentially transfers FAs with no or few double bonds to DGs [29]. Hence, both enzymes directly (DGAT1) or indirectly (PDAT1 via PC) transfer FA from the acyl-CoA-pool to DG to produce TGs. There is no clear cut separation between de novo synthesized FA and membrane derived FA because FAs produced de novo are often acyl-edited by being esterified into PCs with subsequent desaturation.

Analysis of mutants revealed the involvement of membrane remodeling as well as of enzymes in TG metabolism in the response to abiotic stresses [38,39]. It was, e.g., shown that PDAT1 is important for heat-induced TG accumulation [15] and that DGAT1 is involved in TG accumulation in response to freezing [31,40]. TGs accumulated upon *V.l.* treatment in leaves and roots of *pdat1* and *dgat1* mutants similarly to wild type plants (Figure 3), indicating that neither PDAT1 nor DGAT1 are required. Possible scenarios are that PDAT1 and DGAT1 act redundantly or that one enzyme takes over when the other is defective. In addition, other proteins which are present in Arabidopsis have been shown to exhibit DGAT activity and could be involved in the synthesis of TGs upon *V.l.* inoculation [41,42].

Examination of the species composition revealed that upon *V.l.* treatment, roots showed significant increases in levels of all TGs containing FAs with 52 acyl carbons (e.g., 18:x, 18:x, 16:x) or 54 acyl carbons (18:x, 18:x, 18:x), with one to nine double bonds in total. In contrast, little or no increase in TGs containing FAs with only one or two double bonds was detected in leaves. This might be based on a stronger contribution of DGAT1 to TG biosynthesis upon fungal inoculation in roots as compared to leaves. An important involvement of DGAT1 in TG biosynthesis in roots was, e.g., reported for the mutant *sdp1* (SUGAR-DEPENDENT1) [43]. The different TG species composition might also be due to other reasons, e.g., a different lipid composition of the root membranes from which the FAs are released in comparison to leaf membranes. Differences in the lipid composition of leaves and roots are, e.g., based on the enrichment of lipids typical for plastids such as MGDG and DGDG in leaves.

A striking difference was observed in the accumulation of TG 52:9 (18:3, 18:3, 16:3) in response to the different stresses. Basal levels of TG 52:9 were low and increased only slightly in leaves and roots upon *V.l.* or LPS treatment. In contrast, strong increases were observed after treatment with *P. syringae* and induction of AVRRPM1 expression, indicating strong incorporation of galactolipid-derived FA 16:3 in TGs. The latter two conditions resulted in strong damage to leaves, suggesting that remodeling or degradation of cellular membranes and particularly plastid membranes are a major source of FAs for TG synthesis upon *P. syringae* and AVRRPM1 effector-derived stress.

### 4.5. What Is the Relevance of TG Accumulation in Response to Biotic Stress?

In response to stress, the lipid composition of membranes changes. This encompasses the sterol composition and particularly the chain length and desaturation of FAs in membrane lipids. These changes are important mechanisms for adaptation to altered environmental conditions [37]. Upon membrane remodeling, FA can be released. FAs might also originate from damage to membranes due to unfavourable conditions. It is generally accepted that one function of TG synthesis upon stress is to sequester free FAs, since higher levels of free FAs are toxic [5,37]. In addition, TGs accumulating under stress conditions in either cytosolic lipid droplets or plastoglobules might be important to provide energy and building blocks for biosynthetic processes during stress recovery. FAs released from plastid membrane lipids also constitute precursors for signaling molecules such as jasmonates. In this context a plastid-localized heat-inducible lipase (HIL1) was described which preferentially releases 18:3 FA from MGDG [44]. The relevance of TG metabolism in responses to abiotic stress was revealed by the lower stress tolerance of TG synthesis mutants. For instance, the mutants *pdat1* and *hil1* are more sensitive to heat [15,44], and the *dgat1* mutant to freezing and drought [31,40,45].

Less is known on the relevance of TGs in responses to biotic stress. Membranes are involved in the interaction with microbes. Pattern recognition receptors recognizing the presence of MAMPs are localized in or associated with the plasma membrane [46,47]. Examples are the flagellin receptor FLAGELLIN-SENSITIVE 2 (FLS2) and the LPS receptor LORE. Thus, the composition of the membrane might affect the response to pathogens and elicitors. This is especially relevant since the activity of receptors might be dependent on nanodomains. The liquid-ordered phases of nanodomains comprise a particular lipid composition and, e.g., are enriched in sterols and sphingolipids [47,48]. In addition, early responses such as extracellular alkalinization and ion fluxes are associated with the plasma membrane, and membrane dynamics have been shown to regulate the response to flagellin by internalizing the receptor FLS2 [49]. Therefore, membrane remodeling can be expected to be important in responses to biotic stress. Furthermore, release of FAs by lipases or by membrane damage as precursors for molecules with signaling or antimicrobial activities can contribute [7]. Recently, TG levels in potato guard cells have been reported to correlate with the stomatal opening induced by *Phytophthora infestans* [50]. Since the degree of stomatal opening can determine the success of the infecting pathogen, TGs may play a role in pathogen-induced stomatal movement, either as part of plant defense or as part of the manipulation of defense by the pathogen. 

## 5. Conclusions

This study shows that pathogen-derived elicitor and effector molecules are efficient inducers of TG accumulation. As discussed above, the strongest accumulation of TG levels was obtained after induction of AVRRPM1 expression, which resulted in damage and even cell death at later time points. It is reasonable to assume that one possible function of TG synthesis upon this treatment is to reduce the amount of free FAs released by membrane damage or membrane remodeling by incorporating the FAs into TGs. In contrast, *V.l.* and LPS treatment did not induce visible cell damage at the time points tested, and were not associated with a strong increase in levels of TG 52:9, an indicator of plastid lipid damage or remodeling. Thus, the function of TG synthesis in response to *V.l.* and LPS treatment is less clear. This question cannot easily be addressed, since TG synthesis occurs by different pathways, and mutants with defects in the different pathways (such as *pdat1dgat1* double mutants) are not viable. More sophisticated approaches such as the characterization of lines with inducible dysfunction of TG biosynthesis are required to elucidate the relevance of TG synthesis in response to different biotic stresses.

## Data Availability

Not applicable.

## Supplementary Materials

The following are available online at https://www.mdpi.com/article/10.3390/biology10090920/s1, Figure S1: Levels of DG species in roots and leaves after treatment with *V.l.*, Figure S2: Levels of PC species in roots and leaves after treatment with *V.l.*, Figure S3: Levels of MGDG species in roots and leaves after treatment with *V.l.,* Figure S4: Levels of DGDG species in roots and leaves after treatment with *V.l.,* Figure S5: Levels of TG species in roots after treatment with *P. syringae,* Figure S6: TG levels in roots of Dex::avrRPM1 plants after dexamethasone treatment, Figure S7: linear regression analysis for TG levels and the relative amount of *V.l.* DNA in leaves; Table S1: Levels of TGs and *V.l.* DNA in leaves of single plants at time points 1 d to 5 d after treatment of roots with *V.l*.
