# Supplementary material for: Pathogens and Elicitors Induce Local and Systemic Changes in Triacylglycerol Metabolism in Roots and in Leaves of Arabidopsis thaliana
Source: Biology (Basel). 2021 Sep 16;10(9):920. doi: 10.3390/biology10090920 (PMC8465621; doi:10.3390/biology10090920)
Supplement: Supplementary file 1 [file biology-10-00920-s001.zip › biology-1357478-supplementary.pdf]

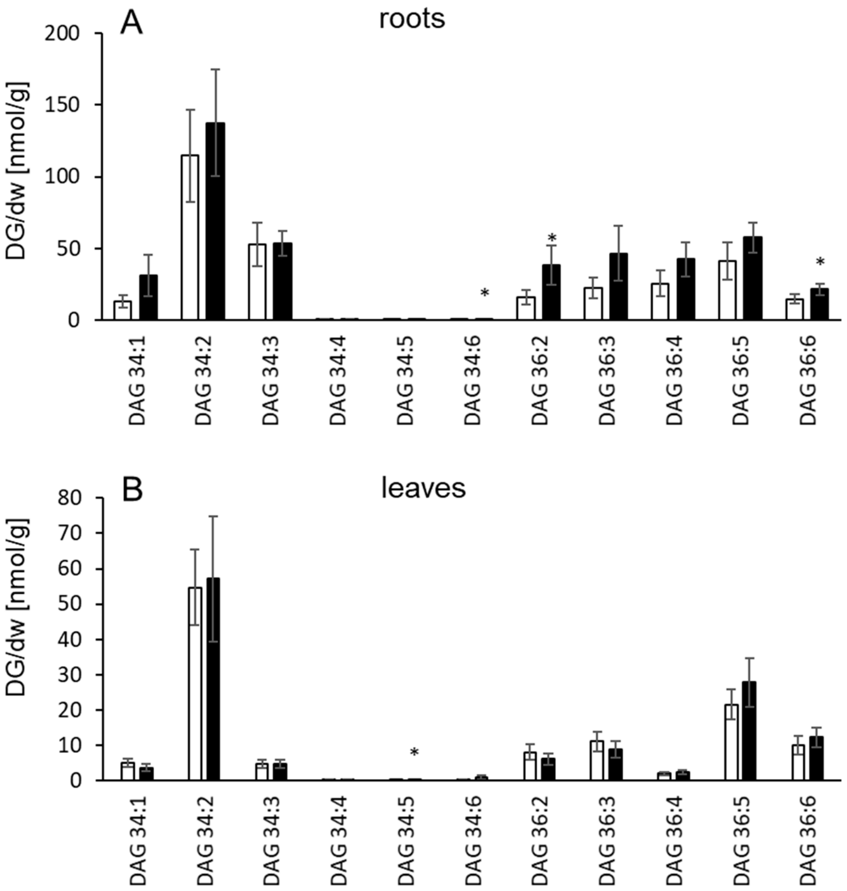


**Supplementary Figure S1.** Levels of different DG species in Col-0 roots (**A**) and leaves (**B**) 3 d after treatment of roots with *V.l.* (black bars) or mock (white bars). DG species are characterized by their total number of acyl carbons and the number of double bonds. DG levels [nmol/g dry weight] shown represent the mean of 5 biological replicates ± sd. Asterisks indicate significant differences of the *V.l.* treated samples compared to the corresponding mock treated sample (* $p < 0.05$).

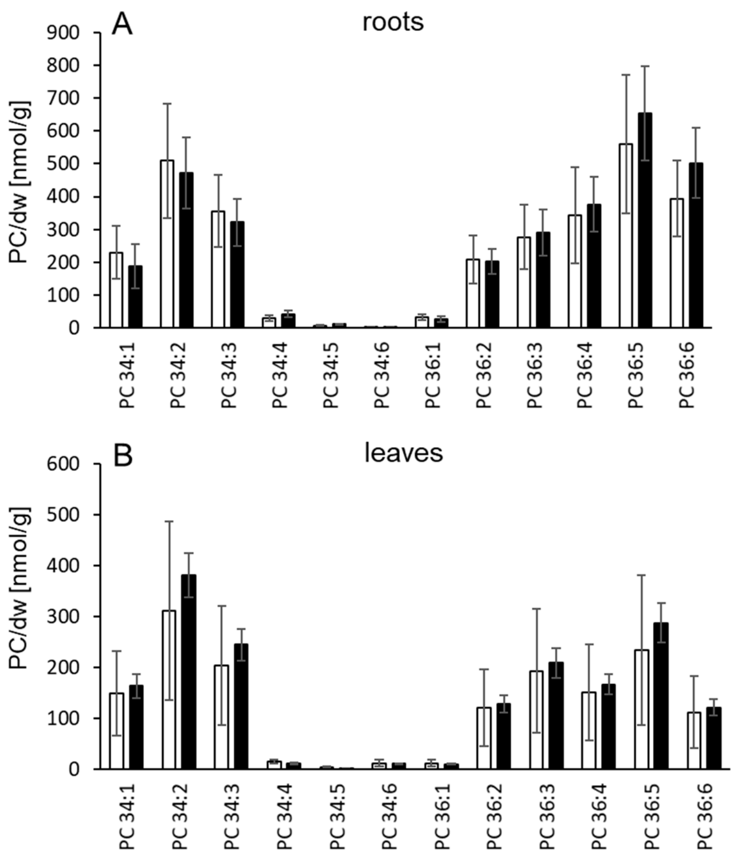


**Supplementary Figure S2.** Levels of different PC species in Col-0 roots (**A**) and leaves (**B**) 3 d after treatment of roots with *V.l.* (black bars) or mock (white bars). PC species are characterized by their total number of acyl carbons and the number of double bonds. PC levels [nmol/g dry weight] shown represent the mean of 5 biological replicates ± sd. No significant differences of the *V.l.* treated samples compared to the corresponding mock treated sample were detected.

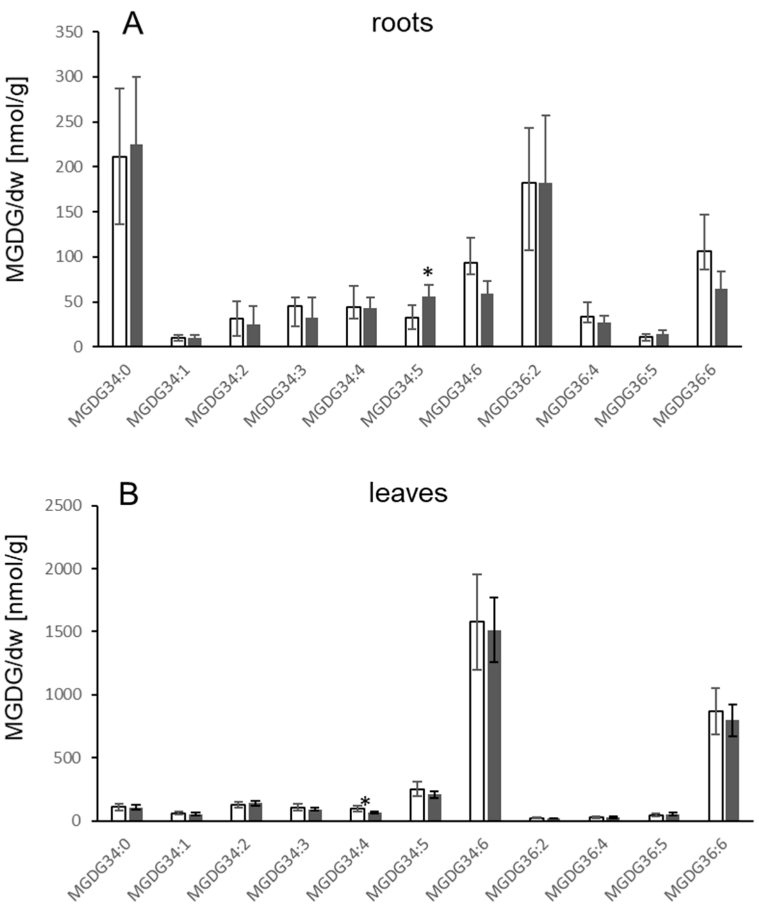


**Supplementary Figure S3.** Levels of different MGDG species in Col-0 roots (**A**) and leaves (**B**) 3 d after treatment of roots with *V.l.* (dark grey bars) or mock (white bars). MGDG species are characterized by their total number of acyl carbons and the number of double bonds. MGDG levels [nmol/g dry weight] shown represent the mean of 5 biological replicates $\pm$ sd. Asterisks indicate significant differences of the *V.l.* treated samples compared to the corresponding mock treated sample (* $p < 0.05$).

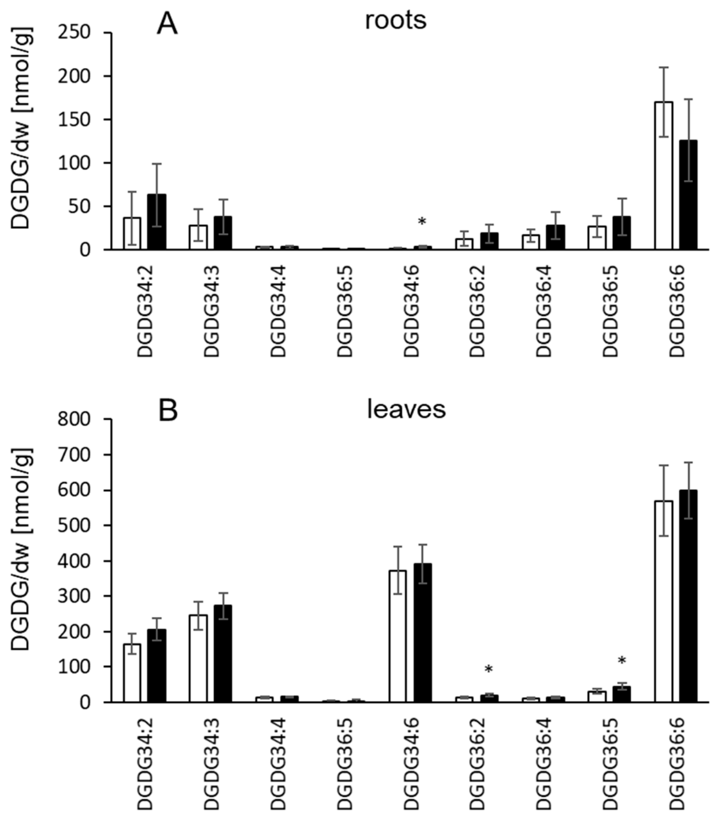


**Supplementary Figure S4.** Levels of different DGDG species in Col-0 roots (**A**) and leaves (**B**) 3 d after treatment of roots with *V.l.* (black bars) or mock (white bars). DGDG species are characterized by their total number of acyl carbons and the number of double bonds. DGDG levels [nmol/g dry weight] shown represent the mean of 5 biological replicates ± sd. Asterisks indicate significant differences of the *V.l.* treated samples compared to the corresponding mock treated sample (* $p < 0.05$).

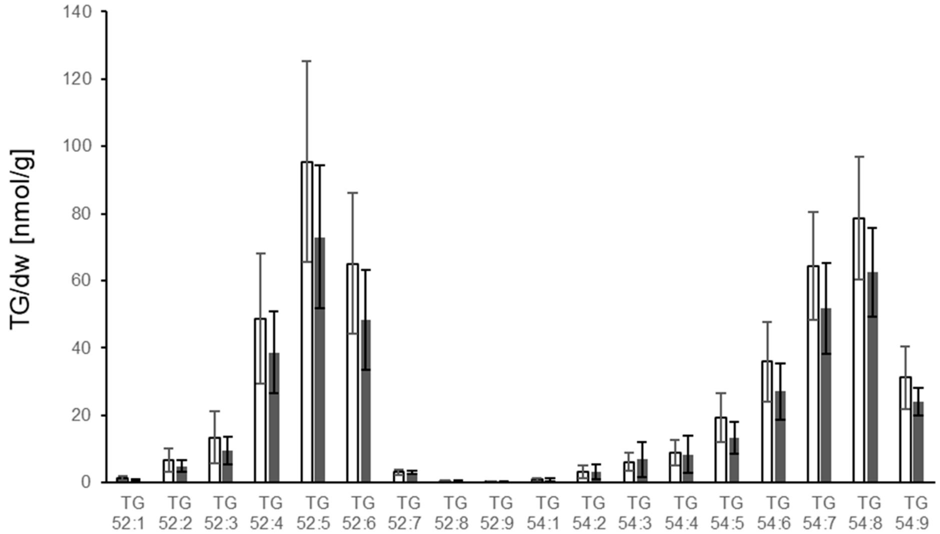


**Supplementary Figure S5.** Levels of different TG species in Col-0 roots 1 d after spraying of leaves with *P. syringae* (dark grey bars) or mock (white bars). TG species are characterized by their total number of acyl carbons and the number of double bonds. TG levels [nmol/g dry weight] shown represent the mean of 5 biological replicates ± sd. No significant differences of the *P. syringae* treated samples compared to the corresponding mock treated sample were detected.

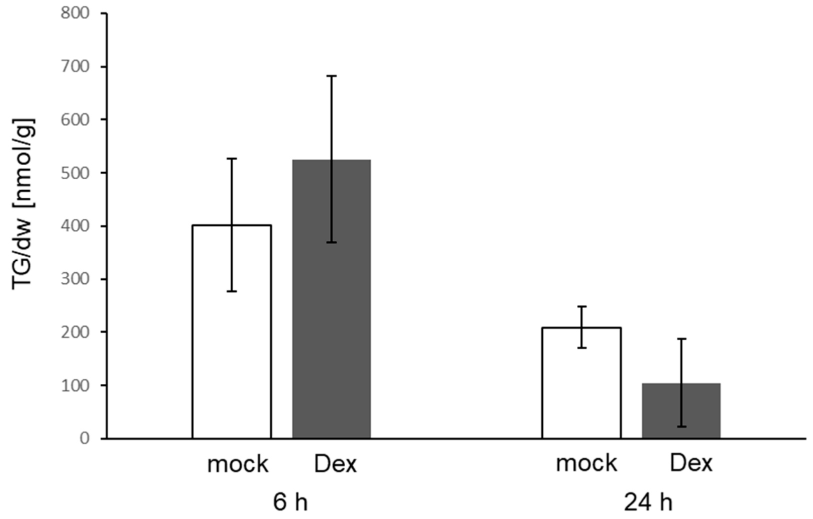


**Supplementary Figure S6.** TGs levels of Col-0 roots 6 h and 24 h after spraying of leaves with dexamethasone to induce expression of AVRRPMI (Dex, dark grey bars) or mock (white bars). Shown is the mean of at least 4 biological replicates ± sd of the sum of TGs [nmol/g dry weight] with 52 and 54 acyl carbon atoms and 1 to 9 double bonds. No significant differences of the dexamethasone treated samples compared to the corresponding mock treated sample were detected.

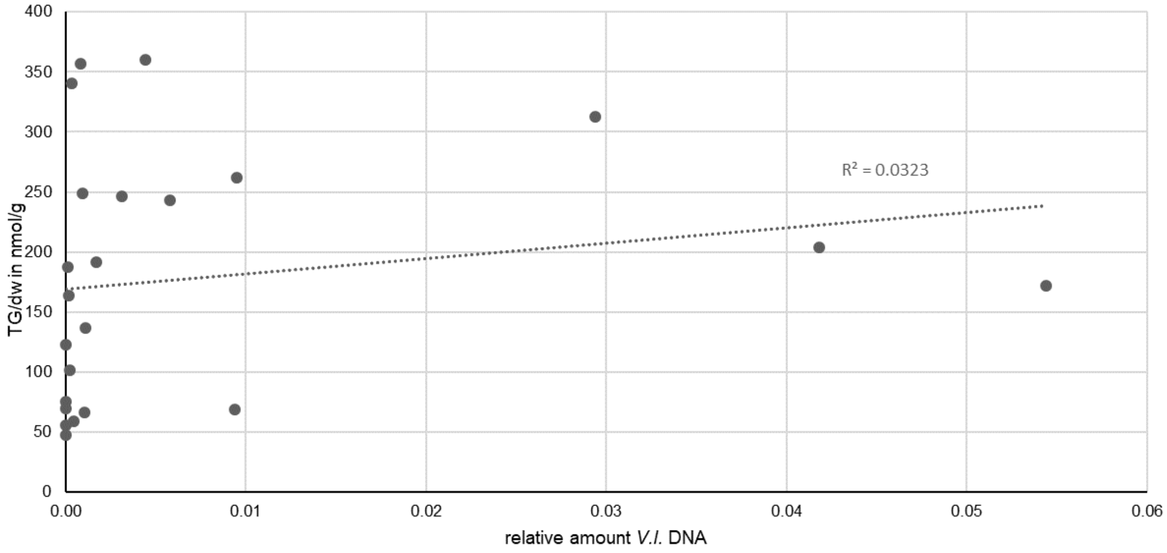


**Supplementary Figure S7.** Linear regression analysis for TG levels and the relative amount of *V.l.* DNA in leaves 1 to 5 d after *V.l.* treatment of roots. R2: coefficient of determination. The analysis is based on the data shown in SupplementaryTable S1.

**Supplementary Table S1**. Analysis of TG levels and relative *V.l.* DNA levels in leaves of single plants at time points 1 d to 5 d.

| | TG | *V.l.* DNA | *V.l.* DNA | *t*-Test |
|---|---|---|---|---|
| Sample | [nmol/g dw] | relative amount | mean | difference to 1 d |
| leaf *V.l.* 1 d | 56 | 0.00000 | 0.00037 | |
| | 59 | 0.00042 | | |
| | 47 | 0.00000 | | |
| | 67 | 0.00106 | | |
| leaf *V.l.* 2 d | 76 | 0.00000 | 0.00234 | 0.435 |
| | 123 | 0.00000 | | |
| | 69 | 0.00936 | | |
| | 70 | 0.00000 | | |
| leaf *V.l.* 3 d | 164 | 0.00014 | 0.00065 | 0.516 |
| | 136 | 0.00108 | | |
| | 102 | 0.00024 | | |
| | 188 | 0.00013 | | |
| | 191 | 0.00167 | | |
| leaf *V.l.* 4 d | 204 | 0.04181 | 0.02265 | 0.107 |
| | 172 | 0.05441 | | |
| | 262 | 0.00949 | | |
| | 360 | 0.00439 | | |
| | 247 | 0.00313 | | |
| leaf *V.l.* 5 d | 341 | 0.00030 | 0.00744 | 0.300 |
| | 249 | 0.00090 | | |
| | 313 | 0.02936 | | |
| | 243 | 0.00580 | | |
| | 357 | 0.00084 | | |

Roots were treated with *V.l.* and leaves were harvested at time points 1 d to 5 d. The amount of *V.l.* DNA is relative to Arabidopsis actin gene determined by qPCR. Included is also the p-value according to Students t-test of the *V.l.* DNA amount of 2 d to 5 d relative to 1 d.
